# Transport Strategy in Patients With Suspected Acute Large Vessel Occlusion Stroke: TRIAGE-STROKE, a Randomized Clinical Trial

**DOI:** 10.1161/STROKEAHA.123.043875

**Published:** 2023-10-06

**Authors:** Anne Behrndtz, Rolf A. Blauenfeldt, Søren P. Johnsen, Jan B. Valentin, Martin F. Gude, Mohammad Ahmad Al-Jazi, Paul von Weitzel-Mudersbach, Boris Modrau, Dorte Damgaard, Kristina Dupont Hougaard, Niels Hjort, Tove Diedrichsen, Marika Poulsen, Marie Louise Schmitz, Marc Fisher, Grethe Andersen, Claus Z. Simonsen

**Affiliations:** Department of Neurology (A.B., R.A.B., D.D., K.D.H., N.H., T.D., M.P., M.L.S., G.A., C.Z.S.), Aarhus University Hospital, Denmark.; Department of Neurology (B.M.), Aarhus University Hospital, Denmark.; Danish Center for Clinical Health Services Research, Department of Clinical Medicine, Aalborg University and Aalborg University Hospital (S.P.J., J.B.V.).; Prehospital Emergency Medical Services, Central Denmark Region (M.F.G.), Goedstrup Hospital.; Department of Neurology (M.A.A.-J., P.v.W.-M.), Goedstrup Hospital.; Department of Neurology, Beth Israel Deaconess Medical Center, Harvard Medical School, Boston (M.F.).; Department of Clinical Medicine, Aarhus University, Denmark (A.B., R.A.B., C.Z.S.).; Department of Neurology, Goedstrup

**Keywords:** hospital, ischemic stroke, odds ratio, patients, thrombolytic therapy

## Abstract

**BACKGROUND:**

When patients with acute ischemic stroke present with suspected large vessel occlusion in the catchment area of a primary stroke center (PSC), the benefit of direct transport to a comprehensive stroke center (CSC) has been suggested. Equipoise remains between transport strategies and the best transport strategy is not well established.

**METHODS:**

We conducted a national investigator-driven, multicenter, randomized, assessor-blinded clinical trial. Patients eligible for intravenous thrombolysis (IVT) who were suspected for large vessel occlusion were randomized 1:1 to admission to the nearest PSC (prioritizing IVT) or direct CSC admission (prioritizing endovascular therapy). The primary outcome was functional improvement at day 90 for all patients with acute ischemic stroke, measured as shift towards a lower score on the modified Rankin Scale score.

**RESULTS:**

From September 2018 to May 2022, we enrolled 171 patients of whom 104 had acute ischemic stroke. The trial was halted before full recruitment. Baseline characteristics were well balanced. Primary analysis of shift in modified Rankin Scale (ordinal logistic regression) revealed an odds ratio for functional improvement at day 90 of 1.42 (95% CI, 0.72–2.82, *P*=0.31). Onset to groin time for patients with large vessel occlusion was 35 minutes (*P*=0.007) shorter when patients were transported to a CSC first, whereas onset to needle (IVT) was 30 minutes (*P*=0.012) shorter when patients were transported to PSC first. IVT was administered in 67% of patients in the PSC group versus 78% in the CSC group and EVT was performed in 53% versus 63% of the patients, respectively.

**CONCLUSIONS:**

This trial investigated the benefit of bypassing PSC. We included only IVT-eligible patients presenting <4 hours from onset and with suspected large vessel occlusion. Lack of power prevented the results from showing effect on functional outcome for patients going directly to CSC.

**REGISTRATION:**

URL: https://www.clinicaltrials.gov; Unique identifier: NCT03542188.

Currently, 2 reperfusion treatment modalities exist for patients with acute ischemic stroke (AIS): intravenous thrombolysis (IVT) and endovascular therapy (EVT) for patients with a large vessel occlusion (LVO). Both treatments are critically time dependent,^1,2^ but EVT is associated with better outcomes than IVT alone for patients with confirmed LVO.^1,3–8^ Benefits of bypassing a primary stroke center (PSC) to arrive faster at a comprehensive stroke center (CSC) with EVT service have been suggested for patients with suspected LVO.^9^ Identifying these patients is recommended,^10^ and prehospital stroke scores have been tested for this purpose. With a sensitivity ranging from 0.50 to 0.67 and a specificity ranging from 0.83 to 0.89, these scales generally perform equally well in finding patients with LVO, but a low accuracy among all tools challenges LVO detection.^11,12^ If patients are suspected for LVO and are ineligible for IVT, guidelines stipulate direct transport to a CSC. The rationale being, EVT remains a possible acute treatment option.^10,13^ The optimal transport strategy for patients with suspicion of LVO and eligibility for IVT remains uncertain. and no significant difference was found in functional outcomes between patients transported to PSC versus CSC in the recent RACECAT trial (Transfer to the Closest Local Stroke Center Versus Direct Transfer to Endovascular Stroke Center of Acute Stroke Patients With Suspected Large Vessel Occlusion in the Catalan Territory) that cluster-randomized patients in a 7-hour window.^14^ The advantage of a bypass transport strategy would be a reduced delay to EVT for patients with confirmed LVO, but the disadvantage would be delay to IVT for patients without LVO. A possible harm for patients with intracerebral hemorrhage (ICH) when subjected to longer transport has also lately been suggested by the RACECAT trialists.

We aimed to investigate the benefits of bypassing the nearest PSC for patients with AIS with suspected LVO, considered eligible for IVT at the prehospital level by measuring functional outcome after 90 days on the modified Rankin Scale (mRS).^15^

## METHODS

### Trial Design

TRIAGE-STROKE (Transport Strategy in Patients With Suspected Acute LVO) was conducted as an investigator-initiated, multicenter, randomized, assessor-blinded trial involving all IVT-eligible patients suspected of LVO in a PSC catchment area. The trial was performed in the Central and Northern regions of Denmark from year 2018 to 2022.

The trial methods have previously been published,^16^ and the protocol and statistical analysis plan are available with the Supplemental Material. The trial was approved by the regional Research Ethics Committee as an acute study. Consent was waived in the acute setting and subsequently obtained from patients or a relative and a trial guardian (supplementary). The study was conducted following good clinical practice E6 guidelines. A steering committee supervised the trial with support from a data monitoring committee. The committee was involved when results from RACECAT were presented and because design, geographic area, and LVO score differed from TRIAGE-STROKE they decided to continue the trial. TRIAGE-STROKE was halted as planned in May 2022 as described in Results.

The trial had no industry involvement but was funded by a nonprofit organization that did not participate in any part of the trial, data analysis, article preparation, or the decision to publish. The report conforms to the recommendations of the CONSORT (Consolidated Standards of Reporting Trials) guidelines. Deidentified data are available from the corresponding author on reasonable request.

### Patients

Patients with acute focal neurological deficits were eligible for inclusion if they were ≥18 years of age, had a Prehospital Acute Stroke Severity (PASS)^17^ score of ≥2, were living independently (prestroke mRS score 0–2), and their symptoms occurred while the patient was in a PSC catchment area (drivetime to PSC was shorter than drivetime to CSC). They had to be candidates for IVT (no oral anticoagulation, recent surgery, or stroke, etc), including being able to arrive at both the CSC and the PSC within 4 hours from stroke onset. Patients were excluded if the stroke occurred inside a hospital or if their life expectancy was below 1 year. Symptom onset was defined as witnessed time of onset or last known well. Decreased consciousness was not an exclusion criterion.

LVO was defined as occlusion of at least one of the following vessels: internal carotid artery, first segment of middle cerebral artery, proximal part of first division of middle cerebral artery, or basilar artery occlusion assessed initially by computed tomography angiography or magnetic resonance angiography, which was accessible at all centers.

The target population was defined before trial commencement as all randomized patients with a final diagnosis of AIS (modified intention to treat) in both transportation arms. The final diagnosis of AIS was validated in all cases by a stroke neurologist.

During the 4-year trial period, the referral 2 PSCs admitted 2491 stroke patients within the 4,5-hour window potentially eligible for screening for LVO symptoms. The shortest road distance between the PSCs and the CSC was 114 km (71 miles) from both PSCs. This corresponds to ≈1 hour of ground transportation in this setting (ambulances are allowed to break traffic law).

### Randomization and Interventions

Prehospital paramedics from 86 ambulance bases (128 ambulances) and 2 helicopter bases (2 helicopters) were trained in recognizing stroke and using the PASS stroke severity score.^17^ PASS was considered positive when at least 2 of the following 3 items were present: eye deviation, arm paresis, and consciousness/aphasia (patients unable to tell own age or current month). PASS was already in use for LVO detection when the trial was initiated. A previous study had shown a sensitivity of 56%, a specificity of 92%, and a positive predictive value of 40% to detect LVO.^18^

Paramedics consulted all patients with suspected LVO with the stroke neurologist at the CSC by telephone conference. Patients considered trial candidates were screened and randomized using the electronic data capture system, which was accessed via a website by the stroke neurologist. Randomization was performed in blocks of 4 and was stratified by age (<65/≥65 years), geographic region, and PASS score (2 versus 3). Patients were randomly assigned in a 1:1 ratio to either direct CSC admission or admission to the nearest PSC.

During the trial period, one of the PSCs was functionally a CSC during daytime. Patients from this center were included only in off-hours (4 pm–8 am) and on weekends.

### Outcomes

All randomized patients were evaluated with mRS on day 90 by trained research personnel blinded to transport strategy (supplementary).^19^ The primary outcome was mRS on day 90. The primary analysis was univariate ordinal logistic regression (shift) to assess functional improvement for the target population (patients with a final AIS diagnosis).

Secondary analyses were shifted for the following subgroups: all randomized patients, all patients with hemorrhages, all patients with stroke mimics, all patients with LVO, all ischemic patients with no LVO, and dichotomized analysis of mRS score of 0 to 2 for all patients with AIS. Excellent outcome (mRS score 0–1), and fair outcome (mRS score 0–3) were defined in post hoc analysis. Other secondary outcomes were time from onset, pick-up, and arrival at first hospital for all patients; and time to IVT (needle time) and EVT (groin puncture) for all treated patients with AIS, successful reperfusion defined as modified Treatment in Cerebral Infarction score of 2b–3, and length of CSC stay. Safety outcomes were severe dependency or death (mRS score of 5–6) in patients treated with EVT. Adverse events and severe adverse events were registered according to good clinical practice guidelines by project nurses during admission and at 90-day examination of the patient (supplementary). To measure prehospital and in-hospital delays, detailed time metrics were collected during the trial by emergency medical staff at ambulances and helicopters, and by stroke clinicians.

For supplementary, we performed a not-planned subgroup analysis where effect was measured in the subgroups of age, sex, diabetes, hypertension, atrial fibrillation, PASS score, use of IVT and EVT, and presence of an LVO.

### Statistical Analysis

We originally estimated that a sample size of 600 patients would provide 80% power to detect a 12% difference between the 2 trial target population groups.

Baseline data for all randomized patients and patients in the target population group was obtained and inspected for imbalances, comparing medians, interquartile ranges (IQRs), and percentages, as appropriate. A multivariate analysis adjusting for age, sex, and prehospital stroke score was performed. The primary target population analysis used univariable ordinal logistic regression comparing the 2 transport arms. The primary effect measure was odds ratio (OR) with a 95% CI for a shift towards a better outcome on the mRS. The Brant and Wald tests were used to show that regression assumption holds true. For the secondary analyses, univariable logistic regression was used to estimate OR for the subgroups, and χ^2^ and *t* tests were used for the dichotomous end points. Secondary efficacy outcomes were not tested for multiple comparisons and the results cannot be used for hypothesis testing or inference.

All analyses were performed using R software version 4.2.1 (R Foundation for Statistical Computing, www.R-project.org).

## RESULTS

### Patient Characteristics

**Figure 1.**
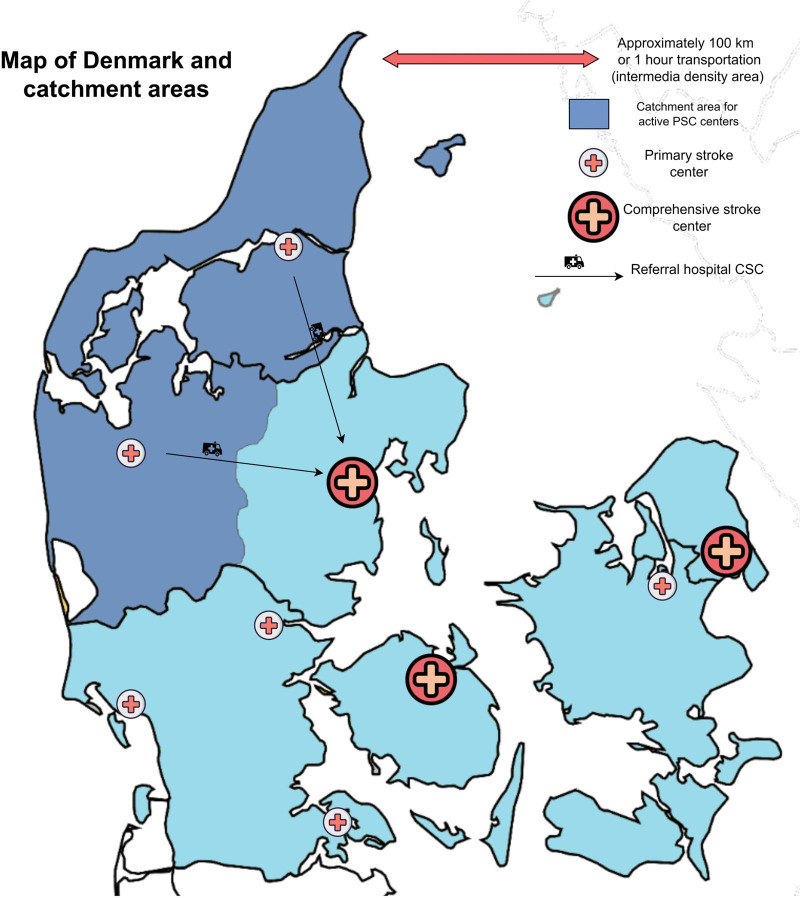
Map of Denmark showing the planned primary and comprehensive stroke centers (CSCs) and the catchment area of the 2 primary stroke centers (PSCs) including during the trial period.

The trial was terminated after 4 years as planned. Lack of funding prevented further extension of the inclusion period. Only 2 out of the planned 6 PSC centers were recruited during the trial period as their corresponding 2 CSCs withdrew agreement to participate after trial initiation (due to the increased burden of accepting the directly admitted patients.) This resulted in poor recruitment (Figure S1). The 2 PSCs had the same corresponding CSC covering an intermediate to rural density catchment area with 887 513 inhabitants (Figure 1).

**Figure 2.**
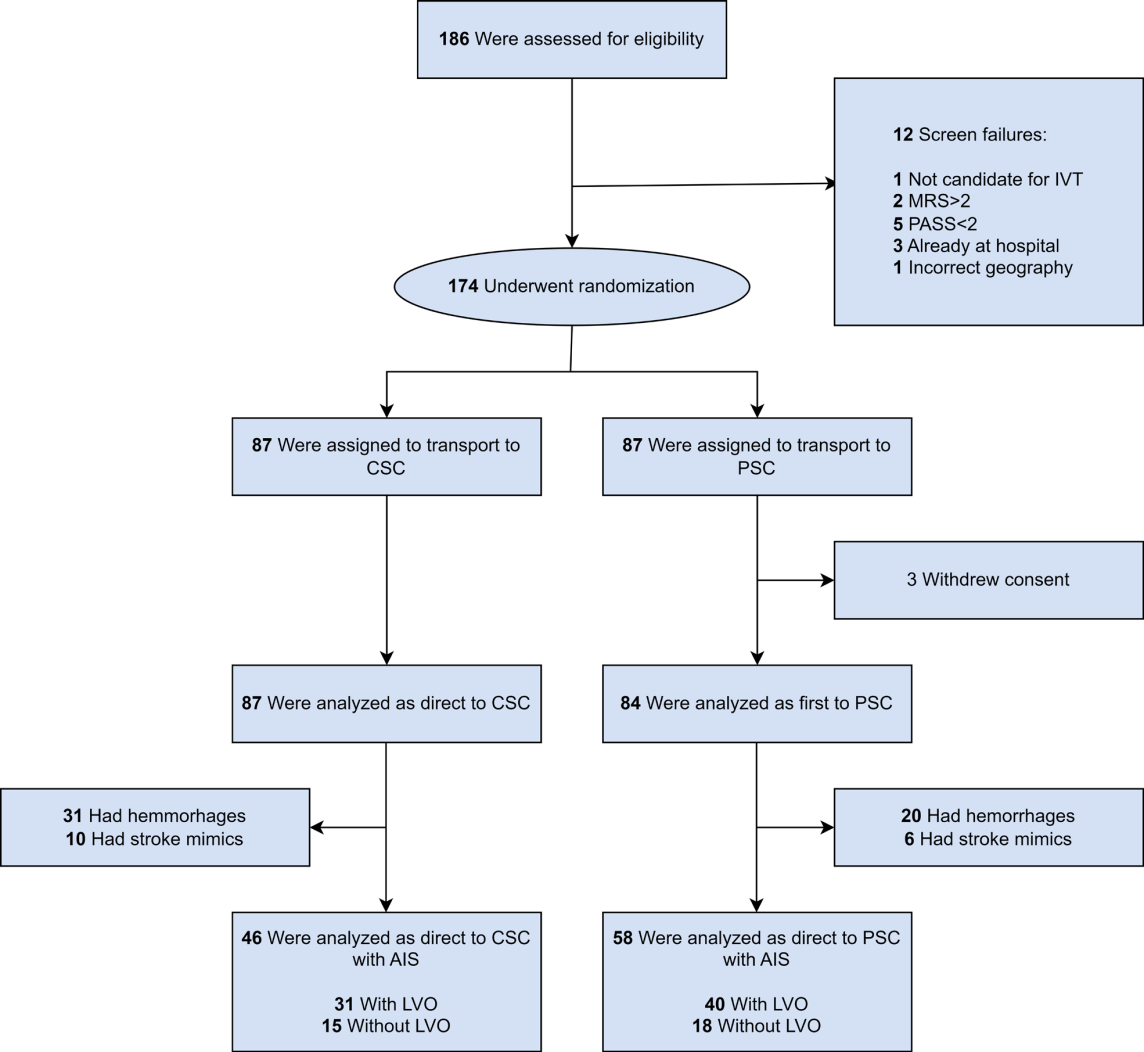
**Randomization, inclusion, and diagnoses of patients.** Patients were randomly assigned in a 1:1 ratio to go either to a primary stroke center (PSC) or directly to a comprehensive stroke center (CSC). Consent was waived in the acute phase. Owing to withdrawal of consent after inclusion, 3 patients underwent randomization but were not included in the trial. All patients were followed, but the primary end point was defined as outcome for all patients with acute ischemic stroke (AIS) including both patients with and without large vessel occlusion (LVO). IVT indicates intravenous thrombolysis; mRS, modified Rankin Scale; and PASS, Prehospital Acute Stroke Severity.

From September 2018 to May 2022, 186 patients were assessed for eligibility, 174 underwent randomization, and 171 gave consent for trial participation; 87 were assigned to transport to the CSC; 84 to a PSC. In the included population, randomization was not respected in 2 patients (1%) because exclusion criteria were revealed after randomization, and another 2 patients (1%) had thrombectomy at the PSC offering daytime thrombectomy. No patients were lost to follow-up and all patients were analyzed after intention to treat principle (Figure 2).

Of the total randomized population, 104 (61%) had AIS, 51 (30%) had ICH, and 16 (9%) had a stroke mimic. Stroke mimics included seizures (N=12), aorta dissection, cervical disc rupture, brain tumor, and hemiplegic migraine (1 each). In the target population with 104 patients with AIS, 71 (68%) had LVO and 33 (32%) had AIS without LVO.

The demographic and clinical characteristics, including prestroke morbidity, stroke severity, and door-to-needle time, were similar in the 2 trial groups, both in the target population and the total randomized population (Table 1). Among all randomized patients, 160 (94%) arrived at the first hospital within 4 hours of onset. Median age in the target population was 74 years (IQR: 66-80), 42 (40%) were females; the median National Institutes of Health Stroke Scale was 16 (IQR, 12–21).

**Table 1.**
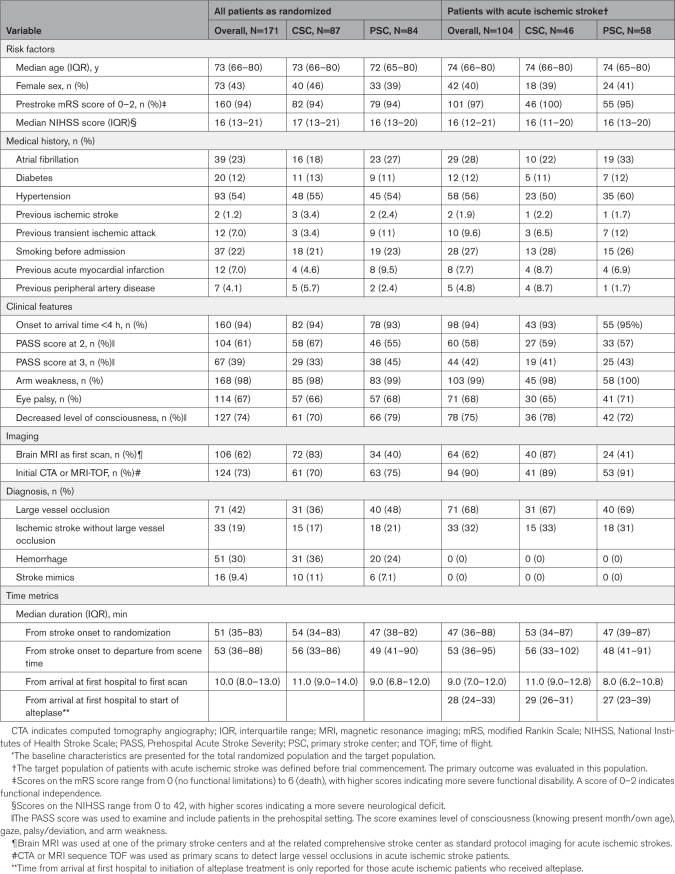
Demographic and Clinical Characteristics of Patients at Baseline^*^

### Primary and Secondary Outcomes

**Figure 3.**
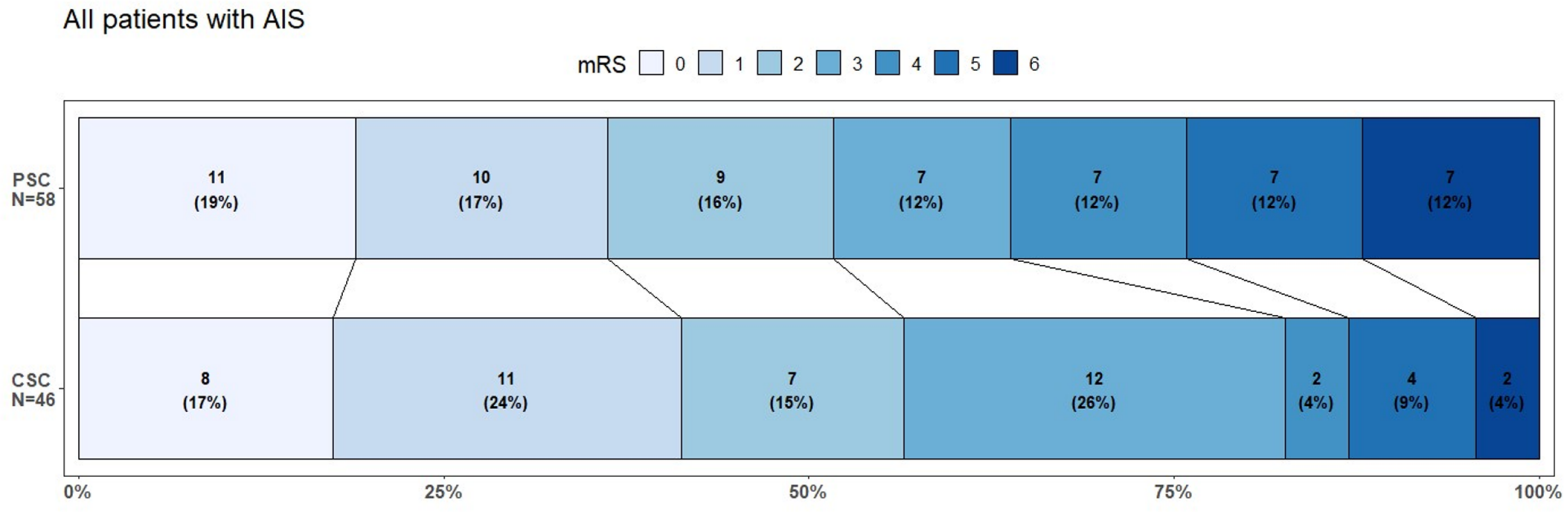
**Distribution of the modified Rankin Scale (mRS) scores at day 90 for all patients with acute ischemic stroke (AIS).** A mRS score of 0 indicates no disability, 1 no clinically significant disability, 2 slight disabilities but independent living, 3, moderate disability but able to walk unassisted, 4 severe disability and unable to walk unassisted, 5 severe disability and bedridden, 6 deaths. CSC indicates comprehensive stroke centers; and PSC, primary stroke centers.

The common OR for improvement measured by mRS at day 90 for all patients with AIS was 1.42; (95% CI, 0.72–2.82) when transported directly to a CSC (Figure 3). The adjusted multivariate analysis revealed an OR of 1.47 (95% CI, 0.74–2.95).

Secondary outcomes showed that for the 71 patients with LVO, OR was 1.41; (95% CI, 0.62–3.24) in favor of going to CSC first; for the 33 patients with AIS and no LVO, the OR was 1.43; (95% CI, 0.43–4.93) in favor of going to a CSC first. For the 51 patients with ICH, the common OR in favor of going to a CSC first was 0.94 (95% CI, 0.34–2.63; Table S1; Grotta bars are shown in Figure S2).

When analyzing dichotomized outcomes of mRS score 0 to 1, we found an OR of 1.24 (95% CI, 0.55–2.76); for mRS score 0 to 2, OR was 1.21 (95% CI, 0.55–2.66); for mRS score 0 to 3, OR was 2.64 (95% CI, 1.06–7.13, *P*=0.034). The percentages of patients with mRS scores 0 to 3 were 83% in the CSC group versus 64% in the PSC group, corresponding to a 19-percentage point absolute risk reduction. This leads to a number needed to transport to CSC of 5 (for patients with AIS) to make 1 more patient ambulatory; the absolute number needed to transport was 8, taking nonischemic diagnoses into account (Table 2).

**Table 2.**
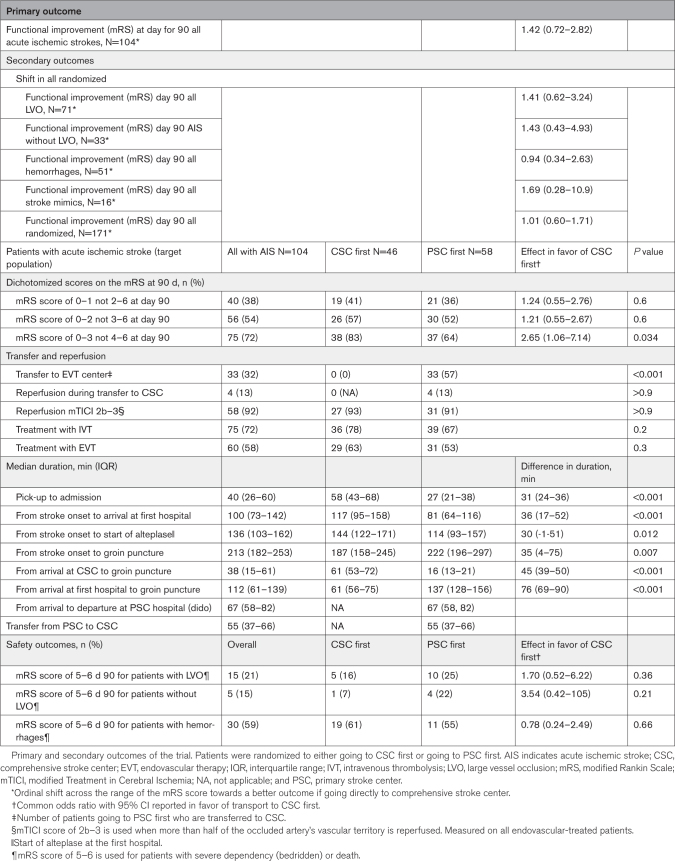
Trial Outcomes^*^

Among the 33 (32%) patients with AIS secondarily transferred from a PSC to a CSC, 4 (13%) were recanalized when arriving at the CSC (2 spontaneously and 2 had IVT). Sixty patients underwent EVT, and reperfusion (modified Treatment in Cerebral Ischemia 2b–3) was obtained in 91% of the PSC group compared with 93% of the patients with EVT in CSC group (*P*>0.9).

The rate of patients treated with IVT when going first to CSC was 78% versus 67% in the group going to PSC first. The rate of patients who underwent EVT was 63% in the first to CSC group and 53% in the PSC group. The differences were not significant (Table S2).

Times from onset to arrival at first hospital was 82 minutes (IQR, 65–115) for patients going first to a PSC and 116 minutes (IQR, 95–150) for patients going directly to a CSC (*P*<0.001). Time from departure from scene to PSC was 27 minutes (19–38); for direct transport to CSC, it was 58 minutes (43–68). Time from onset to needle was 114 minutes (IQR, 93–157) for PSC patients and 144 minutes (IQR, 122–171) for CSC patients (*P*=0.012). Time to groin puncture was 222 minutes (IQR, 196–297) for PSC patients needing transfer and 187 minutes (IQR, 158–245) for CSC patients (*P*=0.007). Hence, onset to groin time for EVT was 35 minutes (*P*=0.007) longer if patients were transported to a PSC first, whereas onset to needle (for IVT) was 30 minutes (*P*=0.012) longer if they were transported directly to a CSC.

Time from arrival at a PSC to departure for EVT treatment (door-in-door-out time) was 67 minutes (IQR, 58–82). When arriving at the CSC, delay to groin puncture was 16 minutes (IQR, 13–21) for patients in the PSC group that were transferred to the CSC versus 61 minutes (IQR, 53–72) for the patients going directly to the CSC. Except for the subgroup females the subgroup analysis showed nonsignificant positive point estimates in all subgroups when going first to CSC (Figure S3).

### Safety Outcomes

Among the total randomized population, OR for receiving a better outcome (shift) was 1.01 (95% CI, 0.60–1.71) when going to a CSC first. The OR of severe dependence or death (mRS score 5–6) at day 90 was 1.72 (95% CI, 0.46–7.27) when transported first to a PSC for patients with LVO, and 3.85 (95% CI, 0.32–210) for patients without LVO when going to PSC first. For patients with hemorrhage, the OR was 0.78 (95% CI, 0.21–2.81) for severe dependency or death when going to a PSC first (Table S3). No imbalances were seen in adverse events (Table S4).

## DISCUSSION

TRIAGE-STROKE randomized patients with clinical signs of LVO eligible for IVT and who had onset of symptoms while located in a PSC catchment area. The trial was carried out in a setting where transport time from the scene to PSC was 27 minutes, transport time from the scene to CSC was 58 minutes, and the transfer time between PSC and CSC was 55 minutes. The study was underpowered and did not have the strength to show an effect on functional outcome at day 90 (OR, 1.42 [95% CI, 0.72–2.82]; *P*=0.3 when transported directly to a CSC).

The Grotta bars indicated benefit in the CSC group, why a post hoc analysis of mRS score of 0 to 3 was made. When dichotomizing the primary outcome for mRS score of 0 to 3 at day 90, the OR was 2.65 (95% CI, 1.06–7.13, *P*=0.034) in favor of going directly to a CSC, suggesting that transport directly to a CSC enhances the probability of becoming ambulatory at day 90. This was a post hoc analysis. We did not adjust for multiple comparisons and the result should be interpreted with caution. Conclusions cannot be made from this.

Transport strategy models to predict optimal transport strategies have investigated thresholds for differences in onset to treatment times, with purpose of achieving the best functional outcome.^20,21^ Based on these, guidelines suggest that for patients with clinical signs of LVO, emergency medical services should bypass the PSC if the bypass time to the CSC is below 30 minutes.^11^ In this study, time from departure from scene to PSC was 27 minutes (19–38); for direct transport to CSC, it was 58 minutes (43–68). This 31-minute time difference renders the results from this trial particularly interesting. Our findings do not challenge the current guidelines to bypass PSC if the increased transport time to CSC is 30 minutes or less. The very efficient workflows at the PSCs shown in the now 2 published randomized controlled trials raise the question if these guidelines also comply with settings where stroke networks operate as efficiently as in these trials.

One randomized controlled trial (RACECAT) has been published previously. The trial cluster-randomized 1401 patients to either PSC/telestroke center or a CSC and analyzed 949 patients with AIS. This trial was neutral with respect to shift in mRS (adjusted common OR, 1.03 [95% CI, 0.82–1.29]).^14^ Nearly a third of the patients in this trial had unknown symptom onset and 76.8% arrived at the CSC within 4 hours. In TRIAGE-STROKE, the corresponding percentage was 94%. In RACECAT there were statistically more patients receiving IVT in the PSC arm (48% versus 60%) and statistically more patients who received EVT in the CSC arm (49% versus 40%). In TRIAGE-STROKE the treatment rates were generally higher, especially for IVT which was 78% for CSC-first patients versus 67% for PSC-first patients and EVT rates were 63% versus 53% respectively (both nonsignificantly different). Some studies have found that the chance of a good outcome decreases exponentially as a function of time in the first hours after LVO onset, and patients presenting early therefore benefit the most.^22^ The difference in designs regarding IVT eligibility between the 2 trials would potentially be a benefit for the patients with LVO going first to CSC in RACECAT. But this benefit is probably outweighed by the delay for IVT among both LVO patients and non-LVO patients who were delayed to the extent that IVT could be given. This is reflected in (low) IVT rates at CSC. The concept in TRIAGE was to investigate patients who were eligible for IVT at both the PSC and the CSC.

TRIAGE-STROKE demonstrated efficient workflows in both arms. The door-in-door-out time at the PSC was 67 minutes and the door-to-groin time was 16 minutes in the PSC arm. Comparing time metrics to RACECAT, patients were delayed approximately half an hour to IVT in both trials when going directly to CSC (30 minutes in TRIAGE-STROKE versus 35 minutes in RACECAT), but delay to EVT was markedly longer in RACECAT (56 minutes versus 35 minutes in TRIAGE-STROKE) for patients going first to PSC. Hence, LVO patients in RACECAT going directly to the CSC were left untreated for a longer time. Time differences in the 2 trials mainly reflect different geographic characteristics (Table S5).

In RACECAT, a trend was observed towards an increased risk of death in patients with ICH (adjusted hazard ratio, 1.21 [95% CI, 0.86–1.70]) if patients were routed directly to CSC. In the present study, no difference in outcome was seen (common OR, 0.94 [95% CI, 0.34–2.63]) for this patient group, however, mortality in patients with ICH in the CSC group was slightly higher compared with the PSC group. This was a nonsignificant difference, but the same trend was seen in RACECAT. This is an issue for further investigation.

The strengths of this study are the valid and individual randomization process, the detailed data collection, and accuracy in collection of prehospital time measures. Furthermore, the study was conducted within the 4.5-hour window of IVT (for patients eligible for IVT) and using a simple stroke severity score (PASS). This study confirmed previous findings that PASS is acceptable for LVO detection with a positive predictive value of 42% in this trial. The tool has been validated to have a PPV of 40 in a previous trial.^12,23^

This trial has several limitations. First, the planned number of included patients was not achieved as described in the preplanned power analysis and the results are underpowered. Second, generalizability beyond the local setting and population in which the trial was conducted may be limited although detailed time metrics were collected to enhance generalizability. The fact that only 2 out of the planned 6 PSCs were recruiting could introduce nonrandom imbalances in this setting. Third, we acknowledge that the treatment rates for IVT are non significantly higher in the group randomized to CSC first and this could reflect in-hospital challenges that are not an issue elsewhere. Guidelines at the centers are the same but a more conservative use of IVT at PSCs should be further investigated as annually reports have shown this tendency^.24^ Fourth, one of the PSCs was functionally a CSC in daytime and the 2 included patients treated with EVT at this PSC potentially contributed to contamination of results.

We suggest to collect more randomized data addressing this topic to sort out the best transport options for patients with suspected LVO.

## CONCLUSIONS

In this trial, where the difference in delay to both IVT and EVT was approximately half an hour, significant benefits in favor of going directly to a CSC were not demonstrated by functional improvement of mRS. Post hoc analysis revealed a statistically significant OR of staying ambulatory (mRS score of 0–3) in the direct-to-CSC group.

## ARTICLE INFORMATION

### Sources of Funding

The trial was funded by the Novo Nordic Foundation (NNF) 170C0029520. Dr Simonsen received grants from the NNF and Health Research Foundation of central Denmark region. Dr Johnsen received grants from Pfizer and NNF.

### Disclosures

Dr Fisher reports employment by Beth Israel Deaconess Medical Center and compensation from Lumosa for consultant services. Dr Blauenfeldt reports compensation from Bayer and Novo Nordisk AS for other services. Dr Johnsen reports compensation from TrygFonden, Phizer, and Bristol Meyers Squibb for consultant services. Dr Simonsen reports employment by Aarhus University Hospital. The other authors report no conflicts.

### Supplemental Material

TRIAGE-STROKE Investigators and Collaborators

Trial Registration and Organization

Supplemental Results

Figures S1–S3

Tables S1–S5

## Supplementary Material


