# Supplementary material for: Transport Strategy in Patients With Suspected Acute Large Vessel Occlusion Stroke: TRIAGE-STROKE, a Randomized Clinical Trial
Source: Stroke. 2023 Oct 6;54(11):2714–23. doi: 10.1161/STROKEAHA.123.043875 (PMC10589426; doi:10.1161/STROKEAHA.123.043875)
Supplement: Supplementary file 1 [file str-54-2714-s001.pdf]

SUPPLEMENTAL MATERIAL

Transport strategy in patients with suspected acute large-vessel occlusion stroke

TRIAGE-STROKE a randomized clinical trial

Anne Behrndtz[17] MD, Rolf A. Blauenfeldt[17] MD, Søren P. Johnsen[2] MD, PhD, Jan B. Valentin[2] MSc, Martin F. Gude[3] MD, Mohammad Ahmad Al-Jazi[4] MD, Paul von Weitzel-Mudersbach[4] MD, PhD, Boris Modrau[5] MD, PhD, Dorte Damgaard[1] MD, PhD, Kristina Dupont Hougaard[1] MD, PhD, Niels Hjort[1] MD, PhD, Tove Diedrichsen[1] MD, Marika Poulsen[1] MD, Marie Louise Schmitz[1] MD, PhD, Marc Fisher[6] MD, Grethe Andersen[1] MD, DMSc, Claus Z. Simonsen[1, 7] MD, PhD

Department of Neurology[1], Aarhus University Hospital, Aarhus, Denmark; Danish Center for Clinical Health Services Research, Department of Clinical Medicine[2], Aalborg University and Aalborg University Hospital; Prehospital Emergency Medical Services, Central Denmark Region[3], Department of Neurology[4], Goedstrup Hospital, Denmark; Department of Neurology[5], Aalborg University Hospital, Denmark; Department of Neurology[6], Beth Israel Deaconess Medical Center, Harvard Medical School, Boston, USA; Department of Clinical Medicine[7], Aarhus University, Aarhus, Denmark.

The supplemental material has been provided by the authors to give readers additional insight of the TRIAGE-STROKE trial. This supplemental material has not been published elsewhere.

Table of content

List of TRIAGE-STROKE Investigators, Collaborators and Affiliations 3
Local principal investigators: 3
Collaborators (trial guardians): 3
Affiliations 3
Trial registration 4
Trial organization 4
Training of Emergency medical staff (EMS): 4
Stroke neurologist training and organization: 4
Information forms -Danish 5
Consent forms -Danish 6
From patient. 6
From a next of kin and a trial guardian 7
Pocket cards (Danish) 7
Structured interview for the modified Rankin scale score (Danish) 8
Description of adverse events (Danish/Table in English) 10
Supplementary Results 11
Figure S1. Inclusion of patients. 11
Figure S2.Grottabars for subgroup diagnoses in the trial. 12
Figure S3. Subgroup analysis 13
Table S1. Ordinal logistic regression table -diagnostic subgroups 14
Length at stay at CSC for patients without LVO 14
Table S2.Treatment rates 14
Table S3. Safety 14
Table S4. Severe adverse events 15
Table S5. Differences between RACECAT and TRIAGE-STROKE 16

List of TRIAGE-STROKE Investigators, Collaborators and Affiliations

Principal Investigator

Claus Z. Simonsen, MD, PhD

Study coordinator

Anne Behrndtz

Local principal investigators:

Birgitte Sandahl, Mohammad Al-Jazi Department of Neurology, Goedstrup

Boris Modrau, Department of Neurology, Aalborg.

Trained stroke neurologists for randomization:Dorte Damgaard, Tove Diedrichsen, Kristina Dupont, Paul von Weitzel-Mudersbach, Niels Hjort, Marika Poulsen, Lise Jeppesen

Trained study nurses: Rikke Bay, Kristina Eiskær, Malene Andersen, Janni Lauridsen, Anne Mette, Anne-Mette Sottrup-Jensen, Nina Jensen, Maiken Hansen.

Other trained medical doctors for including patients and collecting consent: Margret, Jesper Tørring, Anna Ochonske, Morten Stilund, Georgi Sirakov, Goran Bekan, Mayoran Perampalam, Zeinaab Maanaki, Masoud Falah, Janne Mortensen, Søren Due, Niels Degn.

Collaborators (trial guardians):

Department of neuroradiology Goedstrup, Department of neuroradiology, Aarhus, Department of neurosurgery, Aarhus, Department of neuroradiology, Aarhus.

Affiliations

Department of Neurology, Aarhus university hospital, Denmark

Department of neurology, Aalborg university Hospital, Denmark

Department of Neurology, Goedstrup hospital, Denmark

METHODS (supplementary)

Trial registration

Clinical Trials NCT03542188

Ethical Committee (CDR) ID 1-10-72-28-18

Data Committee 1-16-02-957-17

Trial organization

Training of Emergency medical staff (EMS):

Paramedics in the Northern part of Jutland were trained in the full NIHSS and at telephone conference the stroke neurologist PASS was extracted from this when reported in the electronic database. In central Jutland, paramedics were trained in PreSS where Press part 1 is the Cincinnati stroke score plus an option to put in another neurological deficit (e.g., leg palsy, ataxia) and PreSS part 2 is the PASS score.

EMS was trained in concept of the TRIAGE-STROKE trial and in the rationale of obeying randomization. A website and a video was made to enhance dissemination and knowledge of the trial: https://triagestroke.wordpress.com/

Stroke neurologist training and organization:

Prior to the trial the stroke neurologists were trained in using the website-based database. Stroke neurologists at the comprehensive stroke center were trained in screening for patients when the paramedics called from the field. They entered a website with a screening tool and randomized patients if inclusion and exclusion criteria were fulfilled. If they were not fulfilled patients were registered as screen failures. The patients were randomized while the neurologist was on call and the paramedics was informed of which center the patient was randomized to.

All neurologist at the active centers were aware in the study and trained in recognizing symptoms of severe stroke in the purpose of referring patients to the stroke neurologist on call. In addition, Neurologists with competencies in receiving patients for thrombolytic therapy and study nurses were trained in collecting informed consent. And study nurses were trained in collecting data as well.

The patients were allocated to either comprehensive or primary stroke center. The form of transportation device was not dictated. Helicopters were sometimes dispatched after first acute call and were available at scene when randomization was performed. After randomization the prehospital personnel decided the most optimal transportation form. The prehospital times reported are either from the ambulances or the helicopters. When arriving at the hospital guidelines stipulate CTA or MRA for patients with suspected AIS and symptoms of LVO. Patients were treated afterwards according to guidelines.

Information forms -Danish

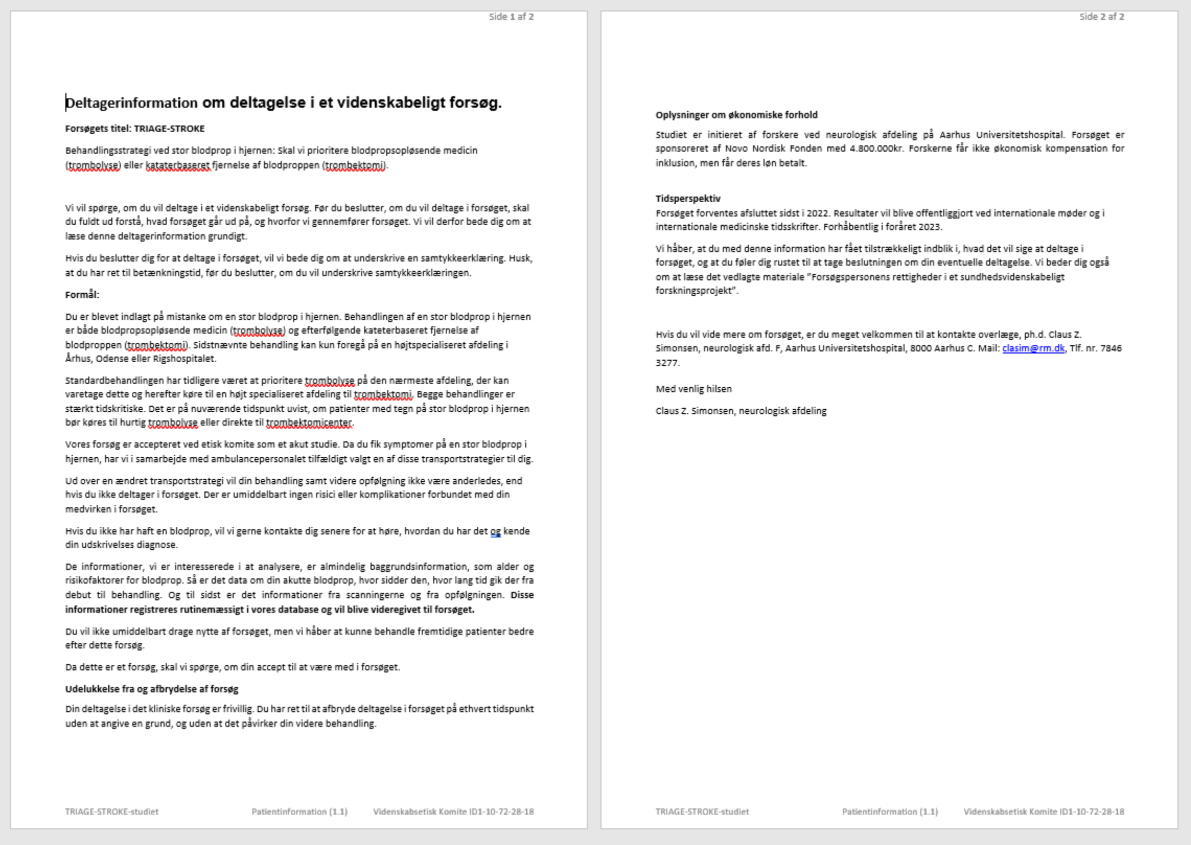

Deltagerinformation om deltagelse i et videnskabeligt forsøg.

Forsøgets titel: TRIAGE-STROKE

Behandlingsstrategi ved stor blodprop i hjernen: Skal vi prioritere blodpropsopløsende medicin (trombolyse) eller kataterbaseret fjernelse af blodproppen (trombektomi).

Vi vil spørge, om du vil deltage i et videnskabeligt forsøg. Før du beslutter, om du vil deltage i forsøget, skal du fuldt ud forstå, hvad forsøget går ud på, og hvorfor vi gennemfører forsøget. Vi vil derfor bede dig om at læse denne deltagerinformation grundigt.

Hvis du beslutter dig for at deltage i forsøget, vil vi bede dig om at underskrive en samtykkeerklæring. Husk, at du har ret til betænkningstid, før du beslutter, om du vil underskrive samtykkeerklæringen.

Formål:

Du er blevet indlagt på mistanke om en stor blodprop i hjernen. Behandlingen af en stor blodprop i hjernen er både blodpropsopløsende medicin (trombolyse) og efterfølgende kateterbaseret fjernelse af blodproppen (trombektomi). Sidstnævnte behandling kan kun foregå på en højtspecialiseret afdeling i Århus, Odense eller Rigshospitalet.

Standardbehandlingen har tidligere været at prioritere trombolyse på den nærmeste afdeling, der kan varetage dette og herefter køre til en højt specialiseret afdeling til trombektomi. Begge behandlinger er stærkt tidskritiske. Det er på nuværende tidspunkt uvist, om patienter med tegn på stor blodprop i hjernen bør køres til hurtig trombolyse eller direkte til trombektomicenter.

Vores forsøg er accepteret ved etisk komite som et akut studie. Da du fik symptomer på en stor blodprop i hjernen, har vi i samarbejde med ambulancepersonalet tilfældigt valgt en af disse transportstrategier til dig.

Ud over en ændret transportstrategi vil din behandling samt videre opfølgning ikke være anderledes, end hvis du ikke deltager i forsøget. Der er umiddelbart ingen risici eller komplikationer forbundet med din medvirken i forsøget.

Hvis du ikke har haft en blodprop, vil vi gerne kontakte dig senere for at høre, hvordan du har det og kende din udskrivelses diagnose.

De informationer, vi er interesserede i at analysere, er almindelig baggrundsinformation, som alder og risikofaktorer for blodprop. Så er det data om din akutte blodprop, hvor sidder den, hvor lang tid gik der fra debut til behandling. Og til sidst er det informationer fra scanningerne og fra opfølgningen. **Disse informationer registreres rutinemæssigt i vores database og vil blive videregivet til forsøget.**

Du vil ikke umiddelbart drage nytte af forsøget, men vi håber at kunne behandle fremtidige patienter bedre efter dette forsøg.

Da dette er et forsøg, skal vi spørge, om din accept til at være med i forsøget.

Udelukkelse fra og afbrydelse af forsøg

Din deltagelse i det kliniske forsøg er frivillig. Du har ret til at afbryde deltagelse i forsøget på ethvert tidspunkt uden at angive en grund, og uden at det påvirker din videre behandling.

Oplysninger om økonomiske forhold

Studiet er initieret af forskere ved neurologisk afdeling på Aarhus Universitetshospital. Forsøget er sponsoreret af Novo Nordisk Fonden med 4.800.000kr. Forskerne får ikke økonomisk kompensation for inklusion, men får deres løn betalt.

Tidsperspektiv
Forsøget forventes afsluttet sidst i 2022. Resultater vil blive offentliggjort ved internationale møder og i internationale medicinske tidsskrifter. Forhåbentlig i foråret 2023.

Vi håber, at du med denne information har fået tilstrækkeligt indblik i, hvad det vil sige at deltage i forsøget, og at du føler dig rustet til at tage beslutningen om din eventuelle deltagelse. Vi beder dig også om at læse det vedlagte materiale "Forsøgspersonens rettigheder i et sundhedsvidenskabeligt forskningsprojekt".

Hvis du vil vide mere om forsøget, er du meget velkommen til at kontakte overlæge, ph.d. Claus Z. Simonsen, neurologisk afd. F, Aarhus Universitetshospital, 8000 Aarhus C. Mail: clasim@rm.dk, Tlf. nr. 7846 3277.

Med venlig hilsen

Claus Z. Simonsen, neurologisk afdeling

Side 1 af 2

Deltagerinformation om deltagelse i et videnskabeligt forsøg.

Til pårørende.

Forsøgets titel: TRIAGE-STROKE

Behandlingsstrategi ved stor blodprop i hjernen: Skal vi prioritere blodpropsopløsende medicin (trombolyse) eller kataterbaseret fjernelse af blodproppen (trombektomi).

Din pårørende har følger efter en neurologisk sygdom. Derfor vil vi spørge dig, om du vil give tilladelse til, om din pårørende vil deltage i et videnskabeligt forsøg. Før du beslutter om du vil give tilladelse, skal du fuldt ud forstå, hvad forsøget går ud på, og hvorfor vi gennemfører forsøget. Vi vil derfor bede dig om at læse denne deltagerinformation grundigt.

Hvis du beslutter dig for at give tilladelse til at din pårørende deltager i forsøget, vil vi bede dig om at underskrive en samtykkeerklæring. Husk, at du har ret til betænkningstid, før du beslutter, om du vil underskrive samtykkeerklæringen.

Formål:

Din pårørende er blevet indlagt på mistanke om en stor blodprop i hjernen. Behandlingen af en stor blodprop i hjernen er både blodpropsopløsende medicin (trombolyse) og efterfølgende kateterbaseret fjernelse af blodproppen (trombektomi). Sidstnævnte behandling kan kun foregå på en højtspecialiseret afdeling i Århus, Odense eller Rigshospitalet.

Standardbehandlingen har tidligere været at prioritere trombolyse på den nærmeste afdeling, der kan varetage dette og herefter køre til en højt specialiseret afdeling til trombektomi. Begge behandlinger er stærkt tidskritiske. Det er på nuværende tidspunkt uvist, om patienter med tegn på stor blodprop i hjernen bør køres til hurtig trombolyse eller direkte til trombektomicenter.

Vores forsøg er accepteret ved etisk komite som et akut studie. Da din pårørende fik symptomer på en stor blodprop i hjernen, har vi i samarbejde med ambulancepersonalet tilfældigt valgt en af disse transportstrategier til din pårørende.

Ud over en ændret transportstrategi vil behandlingen samt videre opfølgning ikke være anderledes, end hvis din pårørende ikke deltager i forsøget. Der er umiddelbart ingen risici eller komplikationer forbundet med din pårørendes medvirken i forsøget.

Hvis din pårørende ikke har haft en blodprop, vil vi gerne kontakte ham/hende senere for at høre, hvordan det går samt kende til udskrivelsesdiagnosen.

De informationer, vi er interesserede i at analysere, er almindelig baggrundsinformation, som alder og risikofaktorer for blodprop. Så er det data om den akutte blodprop, hvor sidder den, hvor lang tid gik der fra debut til behandling. Og til sidst er det informationer fra scanningerne og fra opfølgningen. **Disse informationer vil blive videregivet til forsøget.**

TRIAGE-STROKE-Studiet Pårørendeinformation (1.1) Videnskabs Etisk komite ID 1-10-72-28-18

Side 2 af 2

Din pårørende vil ikke umiddelbart drage nytte af forsøget, men vi håber at kunne behandle fremtidige patienter bedre efter dette forsøg.

Da dette er et forsøg, skal vi spørge, om din accept til at din pårørende kan være med i forsøget.

Udelukkelse fra og afbrydelse af forsøg

Deltagelse i det kliniske forsøg er frivillig. Din pårørende har ret til at afbryde deltagelse i forsøget på ethvert tidspunkt uden at angive en grund, og uden at det påvirker den videre behandling.

Oplysninger om økonomiske forhold

Studiet er initieret af forskere ved neurologisk afdeling på Aarhus Universitetshospital. Forsøget er sponsoreret af Novo Nordisk Fonden med 4.800.000. Forskerne får ikke økonomisk kompensation for inklusion, men får deres løn betalt.

Tidsperspektiv

Forsøget forventes afsluttet sidst i 2022. Resultater vil blive offentliggjort ved internationale møder og i internationale medicinske tidsskrifter. Forhåbentlig i foråret 2023.

Vi håber, at du med denne information har fået tilstrækkeligt indblik i, hvad det vil sige at deltage i forsøget, og at du føler dig rustet til at tage beslutningen om din pårørendes eventuelle deltagelse. Vi beder dig også om at læse det vedlagte materiale "Forsøgspersonens rettigheder i et sundhedsvidenskabeligt forskningsprojekt".

Hvis du vil vide mere om forsøget, er du meget velkommen til at kontakte overlæge, ph.d. Claus Z. Simonsen, neurologisk afd. F, Aarhus Universitetshospital, 8000 Aarhus C. Mail: clasim@rm.dk, Tlf. nr. 7846 3277.

Med venlig hilsen

Claus Z. Simonsen, neurologisk afdeling

TRIAGE-STROKE-Studiet Pårørendeinformation (1.1) Videnskabs Etisk komite ID 1-10-72-28-18

Consent forms -Danish

From patient.

Informeret samtykke til deltagelse i et sundhedsvidenskabeligt forskningsprojekt.

Forskningsprojektets titel: TRIAGE-STROKE

Behandlingsstrategi ved stor blodprop i hjernen: Skal vi prioritere blodpropsopløsende medicin (trombolyse) eller kataterbaseret fjernelse af blodproppen (trombektomi).

Erklæring fra forsøgspersonen:

Jeg har fået skriftlig og mundtlig information og jeg ved nok om formål, metode, fordele og ulemper til at sige ja til at deltage.

Jeg ved, at det er frivilligt at deltage, og at jeg altid kan trække mit samtykke tilbage uden at miste mine nuværende eller fremtidige rettigheder til behandling.

Jeg giver samtykke til, at deltage i forskningsprojektet, og har fået en kopi af dette samtykkeark samt en kopi af den skriftlige information om projektet til eget brug.

Forsøgspersonens navn: ____

Dato: ______________ Underskrift: ____

Ønsker du at blive informeret om forskningsprojektets resultat samt eventuelle konsekvenser for dig?:

Ja _____ (sæt x) Nej _____ (sæt x)

Erklæring fra den, der afgiver information:

Jeg erklærer, at forsøgspersonen har modtaget mundtlig og skriftlig information om forsøget.

Efter min overbevisning er der givet tilstrækkelig information til, at der kan træffes beslutning om deltagelse i forsøget.
Navnet på den, der har afgivet information:

Dato: ______________ Underskrift: ____

Projektidentifikation: Videnskabs Etisk Komite1-10-72-28-18

Patientsamtykke (1.1)

From a next of kin and a trial guardian

Stedfortrædende samtykke til deltagelse i et sundhedsvidenskabeligt forskningsprojekt.

Forskningsprojektets titel: TRIAGE-STROKE

Behandlingsstrategi ved stor blodprop i hjernen: Skal vi prioritere blodpropsopløsende medicin (trombolyse) eller kataterbaseret fjernelse af blodproppen (trombektomi)

Erklæring fra den person, som afgiver stedfortrædende samtykke:

Jeg har fået skriftlig og mundtlig information og jeg ved nok om formål, metode, fordele og ulemper til at give mit samtykke.

Jeg ved, at det er frivilligt at deltage, og at jeg altid kan trække mit samtykke tilbage uden at forsøgspersonen mister sine nuværende eller fremtidige rettigheder til behandling.

Jeg giver samtykke til, at ________________________________ (forsøgspersonens navn) deltager i forskningsprojektet og jeg har fået en kopi af dette samtykkeark samt en kopi af den skriftlige information om projektet til eget brug.

Oplysning om min tilknytning, som pårørende, til forsøgspersonen:

____

Navnet på den person, der giver stedfortrædende samtykke: ______________________

Dato: ____________ Underskrift: ______________________________

Ønskes information om forskningsprojektets resultat samt eventuelle konsekvenser for forsøgspersonen?:

Ja _____ (sæt x) Nej _____ (sæt x)

Erklæring fra den, der afgiver informationen:

Jeg erklærer, at der er afgivet mundtlig og skriftlig information om forsøget.

Navnet på det, der har afgivet information:

Dato: ____________ Underskrift: ______________________________

Stedfortrædende samtykke fra forsøgsværgen (en uafhængig læge):

Dato: ____________ Underskrift: ______________________________

Projektidentifikation: Videnskabs Etisk Komite1-10-72-28-18

Stedfortrædende samtykke (1.1)

Pocket cards (Danish)

Neurologists who receive IVT patients

TRIAGE-STROKE

-Where to go with LVO

Inclusionskriterier:

- **Optageområde Holstebro og Aalborg** (-når der ikke tilbydes trombektomi)
- **Mistanke om stor apopleksi med PreSS del 2 (PASS)≥2**
  (Min. 2 af flg. Sympt.: øjendrejning, armparese, forkert ang. af alder og/eller måned)
- Er **trombolysekandidat** og kan tidsmæssigt modtage trombolyse i Århus.
- **Selvhjulpen** (mRS 0-2).

Eksklusionskriterier:

- **Oplagt anden årsag til symptomer:** (Synkope, kramper, epilepsi, anden medicinsk årsag f.eks hypoglykæmi)
- **Pt. er allerede indlagt**

Ved udskrivelse

- **Noter "Udskrivelse TRIAGE-STROKE" i EPJ/UK**

Kontaktpersoner:

- **Projekt sygeplejerske: Kristina Eiskjær Sørensen (Tlf. 40354814)**
- **Forsøgskoordinator: Anne Behrndtz (Tlf 20784242)**
  Triage-stroke.com

TRIAGE-STROKE

-Where to go with LVO

Visitation ved Forvagt (FV)/Bagvagt(BV)/Trombolysevagt(Tr) :

1. **Ved mistanke om "stor apopleksi" omstil til NVBV**
   Stor apopleksi: min 2 af flg sympt: øjendrejning, armparese, forkert ang. af alder og/eller måned.
2. Ved mistanke om "lille apopleksi" stil igennem til Tr/BV.
3. **Omstilling: #telefonnr.#** (fortæl om pt.)
   Tryk # igen for at viderestille til NVBV.
   (opkaldet kan trækkes tilbage med *)
   Alternativt: bed redderen ringe til NVBV.

Telefonnr neurovaskulære bagvagter:

**NVBV tlf.:**	**20268074**
**Kristina D. Hougaard:**	**30266717**
**Tove Diedrichsen:**	**22547561**
**Paul A. Weizel:**	**42752953**
**Claus z. Simonsen:**	**50240543**
**Dorte Damgaard:**	**22899067**
**Niels Hjort:**	**29936992**
**Marika Poulsen:**	**61681490**
**Lise Jeppesen:**	**30248204**

Stroke neurologist.

TRIAGE-STROKE

-Where to go with LVO

Inclusionskriterier:

- **Optageområde Holstebro og Aalborg** (-når der ikke tilbydes trombektomi)
- **Mistanke om stor apopleksi med PreSS del 2 (PASS)≥2**
  (Min. 2 af flg. Sympt.: øjendrejning, armparese, forkert ang. af alder og/eller måned)
- Er **trombolysekandidat** og kan tidsmæssigt modtage trombolyse i Århus.
- **Selvhjulpen** (mRS 0-2).

Eksklusionskriterier:

- **Oplagt anden årsag til symptomer:** (Synkope, kramper, epilepsi, anden medicinsk årsag f.eks hypoglykæmi)
- **Pt. er allerede indlagt**

Randomisering ved NVBV!:

1. www.ddsc.dk
2. Opret ny patient
3. Tryk på TRIAGE-STROKE
4. Udfyld skema tryk randomiser
5. Fortæl kørselsstrategi til præhospitalet
6. Giv Holstebro/Ålborg besked om kørselsstrategi.
7. Noter **"TRIAGE-STROKE inklusion"** i EPJ
   (kan evt. vente til ankomst)

TRIAGE-STROKE

-Where to go with LVO

Under indlæggelse:

- **Informer om studiet** (se evt. retningslinie for afgivelse af mundtlig deltager information)
- **Indhent samtykke** (TRIAGE-STROKE mappe)
- **Noter i EPJ** om pt er med i TRIAGE-STROKE
  - **"TRIAGE-STROKE informeret samtykke"**
  - **"ønsker ikke at medvirke i TRIAGE-STROKE"**

Ved udskrivelse

- **Noter "Udskrivelse TRIAGE-STROKE" i EPJ/UK**

Kontaktpersoner:

- **Projectsygeplejerske: Kristina Eiskjær Sørensen (Tlf. 40354814)**
- **Forsøgskoordinator: Anne Behrndtz (Tlf. 20784242)**
  Triage-stroke.com

Aalborg

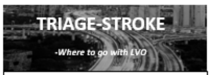

Inklusionskriterier:

1. **Mistanke om stor apopleksi**
   Mindst 2 ud af 3 af disse symptomer:
   - Øjendrejning
   - Armparese
   - Forkert angivelse af alder og/eller måned
2. **Trombektomikandidat** men trombektomi kan ikke tilbydes i Aalborg
3. **Trombolysekandidat** og vil kunne modtage trombolyse inden for 4.5 timer i Århus.
4. **Selvhjulpen** (mRS 0-2).

Eksklusionskriterier:

5. Oplagt anden årsag til symptomer:
   (Synkope, kramper, epilepsi, anden medicinsk årsag f.eks hypoglykæmi)
6. Pt. er allerede indlagt

Inklusion og Eksklusion opfyldt – vend kortet om!

Inklusion til TRAGE-Stroke fortages i Aarhus

1. Opfylder patienten alle inklusionskriterier og ingen eksklusionskriterier da kontakt til trombolysevagt i Aarhus via tryk <u>#23826245#</u> og bede om NVBV (Falck hænger standby i telefon).
   a. Godkender NVBV: Tryk # igen og Falck er forbundet med NVBV
   b. Afviser NVBV: Tryk * og du kan tale med Falck igen og aftale nærmere

   Alternativ. Slutte Falck-samtalen og ring separat til Aarhus og derefter separat tilbage til Falck
2. **Husk kort Visitationsnotat i EPJ**: *"Pt er inkluderet i TRIAGE-STROKE"* hvis NVBV har godkendt inklusion

<u>Kontaktpersoner:</u>

Site-investigator Boris Modrau (Tlf. 61275927)

Projektsygeplejersker:
Ålborg: Nina Jensen (Tlf.97662288)
Århus: Kristina Eiskjær Sørensen (Tlf. 40354814)

Forsøgskoordinator: Anne Behrndtz (Tlf. 20784242)

Triage-stroke.com

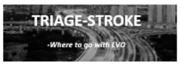

<u>Inclusionskriterier:</u>

- **Optageområde Aalborg** (-når der ikke tilbydes trombektomi i Ålborg)
- **Mistanke om stor apopleksi (PASS≥2)**
  (Se pkt.1 nedenfor)
- Er **trombolysekandidat** og kan tidsmæssigt modtage trombolyse i Århus.
- **Selvhjulpen** (mRS 0-2).

<u>Eksklusionskriterier:</u>

- **Oplagt anden årsag til symptomer:**
  (Synkope, kramper, epilepsi, anden medicinsk årsag f.eks hypoglykæmi)
- **Pt. er allerede indlagt**

<u>Randomisering i Århus!:</u>

1. **Ved mistanke om stor apopleksi og der ikke er mulighed for trombektomi i Ålborg.** <u>Stor apopleksi: min 2 af flg sympt: øjendrejning, armparese, forkert ang. af alder og/eller måned.</u>
2. **Kontakt til Århus: ring til trombolysevagt eller via omstilling:**
3. **Omstilling: <u>#23826245##</u>**
4. Noter **"TRIAGE-STROKE inklusion"** i EPJ hvis patienten randomiseres til kørsel til Ålborg.
   (kan evt. vente til ankomst)

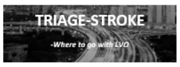

<u>Såfremt patienten FORBLIVER i Ålborg:</u>

- **Informer om studiet** (se evt. retningslinje for afgivelse af mundtlig deltager information)
- **Indhent samtykke** (TRIAGE-STROKE mappe)
- **Noter i EPJ** om pt er med i TRIAGE-STROKE
  - **"TRIAGE-STROKE informeret samtykke"**
  - **"ønsker ikke at medvirke i TRIAGE-STROKE"**

<u>Ved udskrivelse</u>

- **Noter "Udskrivelse TRIAGE-STROKE" i EPJ/UK**

<u>Kontaktpersoner:</u>

- **Site-investigator Boris Modrau (tlf 61275927)**
- **Projektsygeplejersker: Ålborg: Nina Jensen (Tlf.97662288) Århus: Kristina Eiskjær Sørensen (Tlf. 40354814)**
- **Forsøgskoordinator: Anne Behrndtz (Tlf 20784242)**
  Triage-stroke.com

Holstebro/Goedstrup

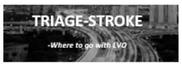

<u>Inclusionskriterier:</u>

- **Optageområde Holstebro og Aalborg** (-når der ikke tilbydes trombektomi)
- **Mistanke om stor apopleksi med PreSS del 2 (PASS)≥2**
  (Min. 2 af flg. Sympt.: øjendrejning, armparese, forkert ang. af alder og/eller måned)
- Er **trombolysekandidat** og kan tidsmæssigt modtage trombolyse i Århus.
- **Selvhjulpen** (mRS 0-2).

<u>Eksklusionskriterier:</u>

- **Oplagt anden årsag til symptomer:**
  (Synkope, kramper, epilepsi, anden medicinsk årsag f.eks hypoglykæmi)
- **Pt. er allerede indlagt**

<u>Randomisering i Århus!:</u>

1. Præhospitalet er informeret om at kontakte Århus ved mistanke om stor apopleksi. <u>Stor apopleksi: min 2 af flg sympt: øjendrejning, armparese, forkert ang. af alder og/eller måned.</u>
2. Såfremt Holstebro kontaktes <u>først</u> bed dem kontakte trombolysevagt i Århus eller via omstilling:
3. **Omstilling: <u>#23826245##</u>**
4. Noter **"TRIAGE-STROKE inklusion"** i EPJ hvis patienten randomiseres til kørsel til Holstebro.
   (kan evt. vente til ankomst)

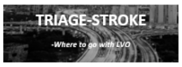

<u>Såfremt patienten FORBLIVER i Holstebro:</u>

- **Informer om studiet** (se evt. retningslinje for afgivelse af mundtlig deltager information)
- **Indhent samtykke** (TRIAGE-STROKE mappe)
- **Noter i EPJ** om pt er med i TRIAGE-STROKE
  - **"TRIAGE-STROKE informeret samtykke"**
  - **"ønsker ikke at medvirke i TRIAGE-STROKE"**

<u>Ved udskrivelse</u>

- **Noter "Udskrivelse TRIAGE-STROKE" i EPJ/UK**

<u>Kontaktpersoner:</u>

- **Projectsygeplejerske: Kristina Eiskjær Sørensen (Tlf. 40354814)**
- **Forsøgskoordinator: Anne Behrndtz (Tlf 20784242)**
  Triage-stroke.com

Structured interview for the modified Rankin scale score (Danish)

FINAL VERSION

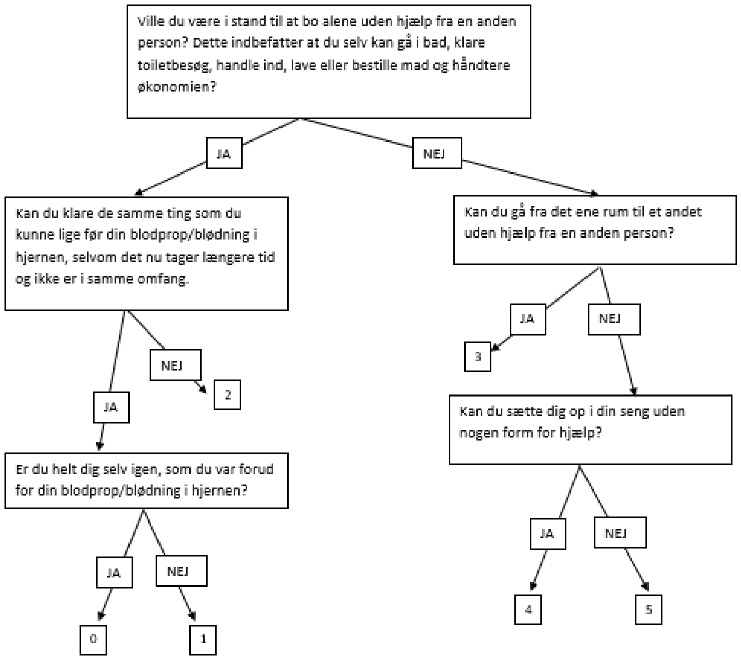

Description of adverse events (Danish/Table in English)

Hændelsesregistrering i databasen

1. Hændelser registreres i DDSC.dk under TRIAGE-STROKE
2. Udfyld skema i DDSC.dk (såfremt der er systemnedbrud benyt papir)
3. Se klassificering af forventede hændelser bagerst i dette afsnit.
4. Når denne sendes genereres mail til sponsor. Denne vurderer herefter hændelsen

Klassificering af hændelser i TRIAGE-STROKE

Definitioner:

AE= Adverse event

Enhver uønsket hændelse hos en patient eller forsøgsperson i et klinisk forsøg med afprøvning af ny metode, uden der nødvendigvis er en sammenhæng mellem denne behandling og den uønskede hændelse.

SAE=Serious adverse events

En hændelse, der medfører:

1) Død
2) Alvorlig forringelse af helbredeet, som forårsager livstruende sygdom eller skade på kroppen eller kropsfunktioner, eller hospitalsindlæggelse eller forlængelse af hospitalsophold, eller et medicinsk eller kirurgisk behandling er nødvendig for at undgå ovenstående, eller
3) Fosterdød, en medfødt anomali eller misdannelse hos fosteret eller anden negativ påvirkning af fosteret.

Eller

Næsten-hændelser
Alvorlige hændelser også den situation, hvor det under 1.-3. nævnte kunne være sket, hvis ikke der var blevet grebet ind eller forholdene havde været mindre gunstige. Det er hændelser, som lægemiddelstyrelsen betegner som **næsten-hændelser**. Herudover kan en alvorlig hændelse være en hændelse, der skyldes **unøjagtige eller mangelfulde resultater** fra diagnostisk udstyr, der resulterer i fejldiagnose, forsinket diagnose eller forsinket eller forkert behandling og de ovenstående hændelser indtræffer.

SAE begrebet i TRIAGE-STROKE

Apopleksi er en akut og livstruende tilstand med stor risiko for komplikationer og forværring i den akutte fase. Naturforløbet ved apopleksi er forbundet med stor risiko for komplikationer, so kan omfatte: Forværring af symptomer/"apopleksi i progression", symptomatisk hæmorrhagisk transformation, dysfagi, kardielle arytmier, pneumoni og andre alvorlige infektioner. Disse relationer er alle relateret til årsagen til indlæggelsen og er forventelige se index apopleksi. Disse events skal såfremt de efter specialistvurdering/investigators vurdering er en del af naturforløbet ved apopleksi ikke indrapporteres akut til sponsor.

Indrapportering:

Sitet indrapporterer SAE elektronisk i ddsc.dk og der genereres straks e-mail til sponsor og forsøgskoordinator.

Forventelige SAEér gennemgåes og en gang hver 3. måned vurderes om dette svarer til det forventelige. Disse indrapporteres til Videnskabs etisk komite (VEK) en gang årligt.

Ikke forventede SAEér indrapporteres af sitet til sponsor inden for 24 timer og denne underretter VEK indenfor 7 dage.

Forventede SAE

Patient Population	Event	Frequency	Mitigation/treatment
TIA and AIS	Recurrent stroke	5-10% at 90 days[i]	Standard medical treatment, IVT/EVT
AIS	Symptomatic ICH	8%[ii]	Antitrombotic/anticoagulation therapy cessation. Antifibrinolytic agent. Blodpressure control.
AIS,ICH	Early (<24hours) and late (>24hours) deterioration (ΔNIHSS>2 or death)	7-22%[iii]	Standard treatment according to etiology
AIS,ICH	Seizures	9%[iv]	Antiepileptic treatment according to in hospital SOP
AIS,ICH	Aspiration pneumonia	5-20%[v]	Antibiotic treatment according to inhospital SOP
AIS,ICH	Urinary tract infections	12%[vi]	Antibiotic treatment according to inhospital SOP
AIS,ICH	Severe infections	7-13%[vii]	Antibiotic treatment according to inhospital SOP
AIS,ICH	Cardiac arrythmia	29%[viii]	Depending on type of arrythmia. Treatment according to cadio.dk
AIS,ICH	Acute myocardial infarction	1-6%[ix]	Treatment according to cadio.dk and conference with cardiologist
AIS,ICH	Deep venous thrombosis (DVT)/pulmonary embolism(PE)	DVT:8%[x]<br>PE: 1%[xi]	Treatment according to cadio.dk
AIS,ICH	Falls (inhospital-phase)	14-65%[xii,xiii]	Treatment depends on type of injury see in hospital SOP
AIS,ICH	Mortality	AIS: 10-20%[xiv]<br>ICH:30%[xv]	

TIA= transitory iscemic apoplexy

AIS= Acute ischemic apoplexy

ICH= Intracerebral hemorrhage

IVT= Intravenous thrombolysis

EVT= Endovascular treatment

[i] Wang Y et al. Clopidogrel with Aspirin in Acute Minor Stroke or Transient Ischemic Attack. NEJM. 2013; 369:11-19

[ii] Berkhemer OA. A Randomized Trial of Intraarterial Treatment for Acute Ischemic Stroke. NEJM. 2015; 372:11-20

[iii] Kwan j et al: early neurological detoriation in acute stroke: clinical characteristics and impact on outcome Journal of the Association of Physicians, 2006, Sept; 99:9: 625-633

[iv] J. Nuyen, F.G. Schellevisa, A. William, et al.Comorbidity was associated with neurologic and psychiatric diseases: a general practice-based controlled study J Clin Epidemiol, 59 (2006), pp. 1274-1284

[v] Hannawi Y et al. Stroke-Associated Pneumonia: Major Advances and Obstacles. Cerebrovasc Dis. 2013;35:430-443

[vi] Bogason E. Urinary Tract Infections in Hospitalized Ischemic Stroke Patients: Source and Impact on Outcome. 2017;9(2):e1014. doi:10.7759/cureus.1014

[vii] BergerB. Epidemiologic features, risk factors, and outcome of sepsis in stroke patients treated on a neurologic intensive care unit. Journal of Critical care. 2013;29(2)

[viii] Daniele O et al. Stroke and cardiac arrhythmias. J Stroke Cerebrovasc Dis. 2002 jan-feb;11(1):28-33

[ix] Johnston KC et al. Medical and neurological complications of ischemic stroke: experience from the RANTTAS trial. RANTTAS Investigators. Stroke. 1998;29(2):447

[x] Bembenek J. Early stroke-related deep venous thrombosis: risk factors and influence on outcome. J thromb Thrombolysis. 2011 Jul; 32(1):96-102

[xi] Pongmoragot J. et al. Pulmonary Embolism in Ischemic Stroke: Clinical Presentation, Risk Factors, and Outcome. J Am Heart Ass. 2013 Dec;2(6)

[xii] Davenport RJ, Dennis MS, Wellwood I, Warlow CP. Complications after acute stroke. Stroke. 1996; 27: 415–420

[xiii] Teasell R, McRae M, Foley N, Bhardwaj A. The incidence and consequences of falls in stroke patients during inpatient rehabilitation: Factors associated with high risk. Arch Phys Med Rehabil. 2002; 83: 329–333.

[xiv] Teasell R, McRae M, Foley N, Bhardwaj A. The incidence and consequences of falls in stroke patients during inpatient rehabilitation: Factors associated with high risk. Arch Phys Med Rehabil. 2002; 83: 329–333.

[xv] Lichtman JH et al. 30-Day Mortality and Readmission after Hemorrhagic Stroke among Medicare

Supplementary Results

Figure S1. Inclusion of patients.

Shows the inclusion rates per month during the trial. The trial was not paused at any time during the trial period and inclusion was relatively stable during the whole period.

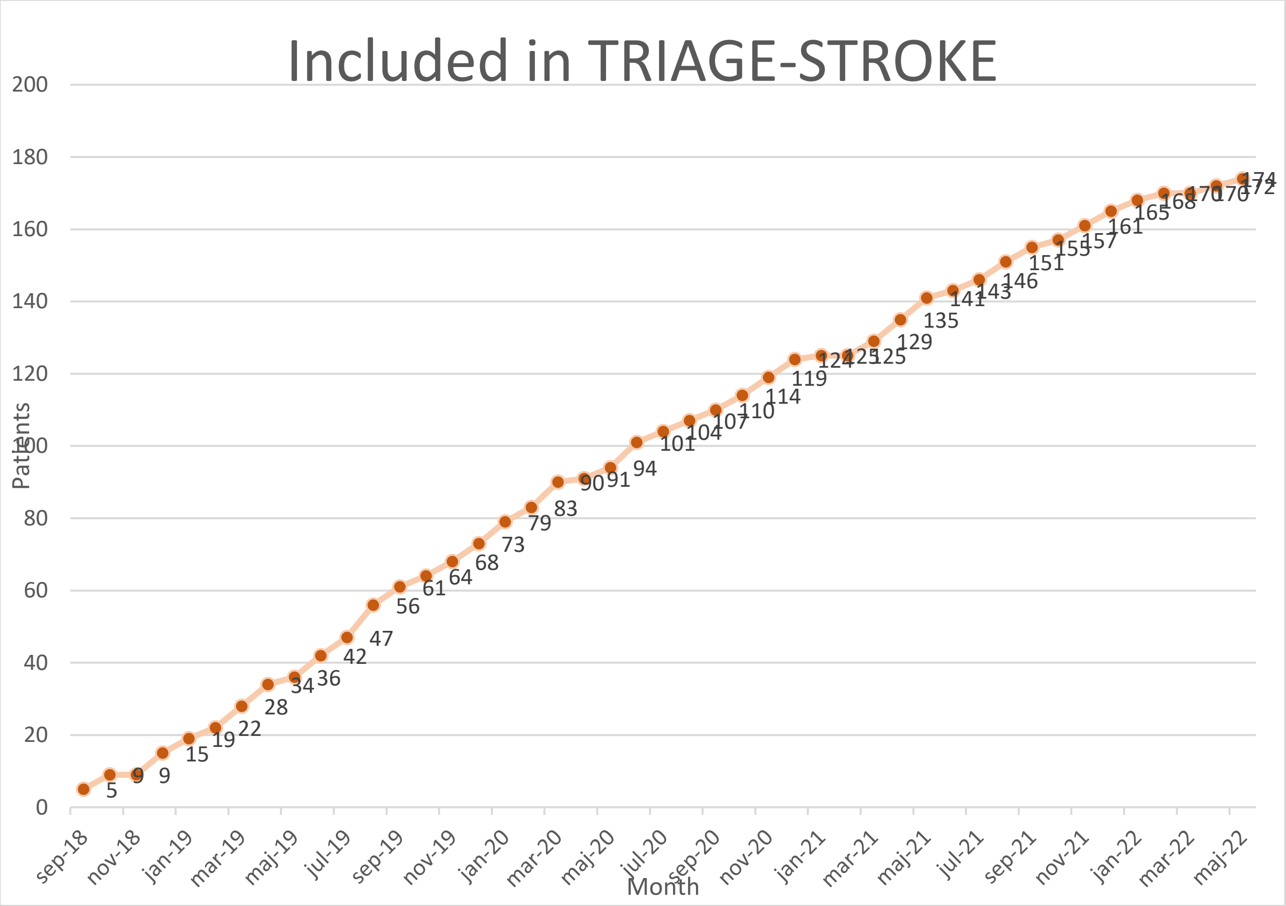

Figure S2.Grottabars for subgroup diagnoses in the trial.

Distribution of the Modified Rankin scale scores at day 90 for patients with large vessel occlusion (LVO), no LVO, Intracranial hemorrhages (Hemorrhages) and with stroke mimics (Mimics).
A modified Rankin Scale score of 0 indicates no disability, 1 no clinically significant disability, 2 slight disabilities but independent living, 3, moderate disability but able to walk unassisted, 4 severe disability and unable to walk unassisted, 5 severe disability and bedridden, 6 deaths.

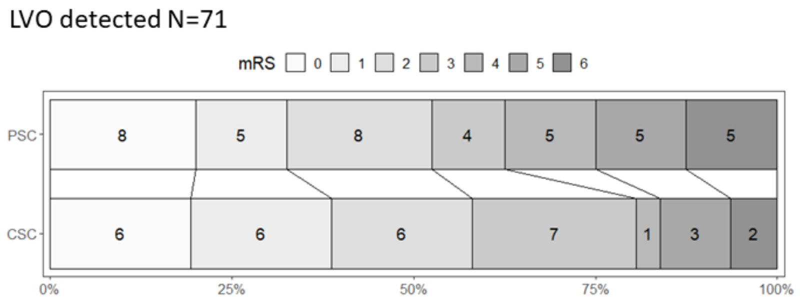

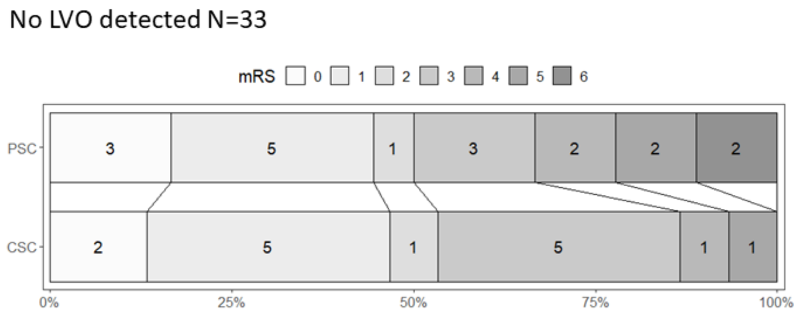

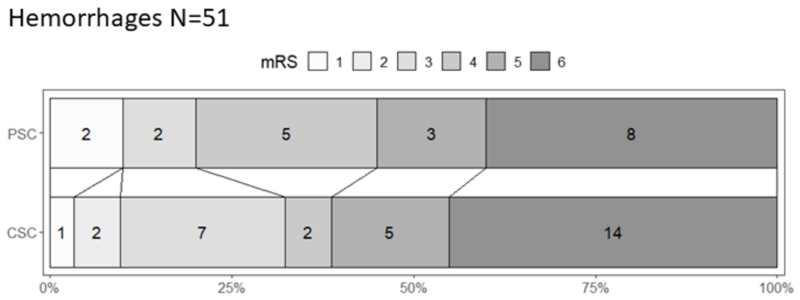

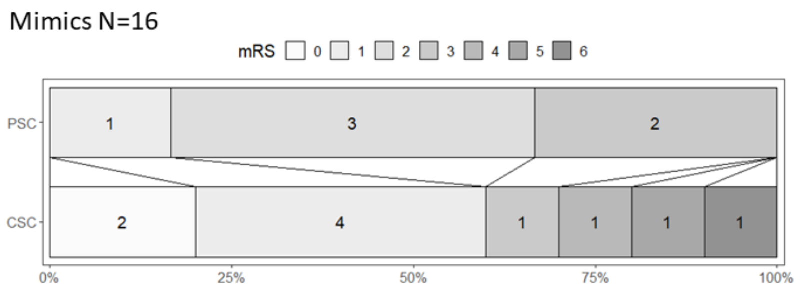

Figure S3. Subgroup analysis

Figure S3. Subgroup analysis. We analyzed the common odds ratio for shift (with ordinal logistic regression) towards a better functional outcome if transported directly to CSC.

Subgroup	PSC (n)	CSC (n)	OR (95%CI)
More than 75 yr	29	22	1.56 (0.56-4.42)
Less than 75 yr	29	24	1.18 (0.46-3.09)
Females	24	18	0.92 (0.31-2.71)
Males	34	28	1.82 (0.75-4.48)
Diabetes	7	5	5.61 (0.59-76.7)
No Diabetes	51	41	1.34 (0.65-2.78)
Hypertension	35	23	1.79 (0.71-4.60)
No Hypertension	23	23	0.96 (0.34-2.68)
Atrialfibrillation	19	10	1.57(0.41-6.14)
No Atrialfibrillation	39	36	1.32(0.59-2.94)
PASS 2	33	27	1.15 (0.47-2.83)
PASS 3	25	19	1.86 (0.65-5.43)
Use of IVT	39	36	1.38 (0.62-3.08)
No use of IVT	19	10	1.24(0.33-4.65)
Use of EVT	31	29	1.26 (0.51-3.09)
No use of EVT	27	17	1.70(0.59-5.04)
LVO	40	31	1.41 (0.62-3.24)
AIS without LVO	18	15	1.43(0.43-4.93)
**All AIS**	**58**	**46**	**1.42**

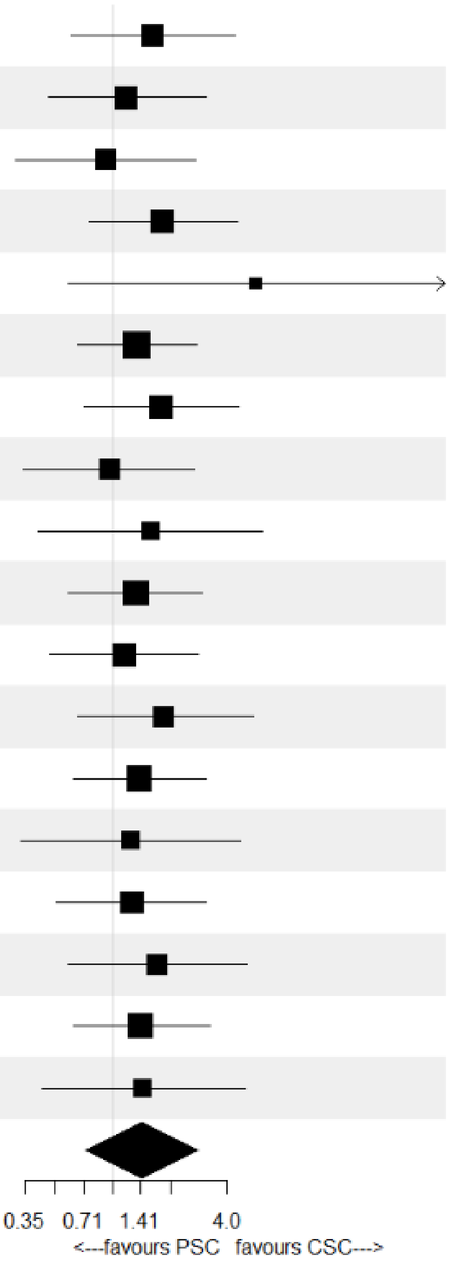

Table S1. Ordinal logistic regression table -diagnostic subgroups

Diagnostic group	N	OR*	(95% CI)	p-value
All AIS	104	1.42	(0.72-2.82)	0.31
LVO	71	1.41	(0.62- 3.24)	0.41
AIS without LVO	33	1.43	(0.43-4.93)	0.56
Hemorrhages	51	0.94	(0.34-2.63)	0.91
Mimics	16	1.69	(0.28-10.9)	0.57
All randomized	171	1.01	(0.60-1.71)	0.97

* Common odds ratio with 95% confidence interval reported in favor of transport to CSC first. Reported as ordinal shift across the range of the modified Rankin Scale score (mRS) toward a better outcome if going directly to comprehensive stroke center
OR = Odds Ratio, CI = Confidence Interval

Length at stay at CSC for patients without LVO

Median length of stay at CSC was one day (IQR 1.00-2.00) for patients with stroke mimics, intracerebral hemorrhages and AIS without LVO.

Table S2.Treatment rates

**Variable**	**Patients with Acute Ischemic Stroke †**		
	Overall, N=104	CSC, N=46	PSC, N=58
Treatment with IVT only	29(28)	14(30)	15(26)
Treatment with EVT only	14(13)	7(15)	7(12)
Treatment with EVT and IVT	46(44)	22(48)	24(41)

Table S2. Individual treatment rates in the AIS population

Table S3. Safety

**Table S4. Safety outcomes: severe dependency or death**	
**Severe independency or death (mRS 5-6)**	**OR (95% CI)***
OR for mRS 5-6 of patients with Large Vessel Occlusions (LVO)	1.72(0.46-7.27)
OR for mRS 5-6 of patients without LVO	3.85(0.32-210)
OR for mRS 5-6 of patients with Intracranial hemorrhages	0.78 (0.21-2.81)

*Common odds ratio (OR) for severe independency or death measured as a dichotomous outcome for modified Rankin Scale Score of 5 (severe dependency) or 6(death)

Table S4. Severe adverse events

Table S3 .Severe Adverse Events			
	**CSC group, N=87**	**PSC group, N=84**	**p-value**
**Expected severe adverse events N (%)**	14 (16)	12 (14)	0.7
Not expected severe adverse events N(%)	4 (5)	6 (7)	0.7
Adverse Events	0(0)	0(0)	1
**Expected severe adverse events***			**Expected rate**
Deaths after intracranial hemorrhages	10 (11)	7 (8)	30%
Deaths after acute ischemic stroke	1 (1)	1 (1)	10-20%
Death after IVT and EVT†	0(0)	1 (1)	1-10%
Aspiration pneumonia**	0(0)	1 (1)	5-20%
Pneumonia	1 (1)	1 (1)	7-13%
Stroke or TIA‡ within 3 months of randomization	2 (2)	1 (1)	5-10%
**Unexpected severe adverse events**§			
Lung edema	0(0)	1 (1)	
Infection, unspecified	0(0)	1 (1)	
Stroke mimicry	0(0)	1 (1)	
Pacemaker	1 (1)	0(0)	
Benign colon tumor operation	1 (1)	0(0)	
Arthrocentesis	0(0)	1 (1)	
Nasogastric tube not placed correctly	0(0)	1 (1)	
Aortic dissection	1 (1)	0(0)	
Pulmonary embolism	1 (1)	0(0)	
Alcohol intake and fainting	0(0)	1 (1)	

Expected adverse events registered by study nurse from files during first admission or at 3 month evaluation.
*Expected severe events were defined prior to the trial please see previous table I supplementary.
†Death after treatment with intravenous thrombolysis (IVT) or endovascular treatment (EVT) was not specified prior to the trial but was considered included in the death after acute ischemic stroke.
‡ Transitory ischemic attack (TIA).
§ Unexpected severe adverse events was not events not defined as expected before trial initiation.
**Detected and diagnosed by physician (clinical, radiological or initiation of antibiotics within 48 hours)

Table S5. Differences between RACECAT and TRIAGE-STROKE

**Table S5. Differences between RACECAT and TRIAGE-STROKE**		
	RACECAT	TRIAGE-STROKE
**Inclusion criteria**		
Geografical area	Catalonoia	Denmark (western and northern part)
Arrival within	7 hours of onset	4 hours of onset
Prehospital assessment	RACE	PASS
Assessment for randomization	Paramedic	Stroke-neurologist
**Randomization**	Cluster	Electronic data capture system
**Results**		
Baseline		
Included	1369	171
Target (AIS patients (N))	949 (69)	104 (61)
LVO (% of all)	636 (46)	71(42)
AIS without LVO(% of all)	313(23)	33(19)
Hemorrhages(% of all)	314 (23)	51(30)
Mimics(% of all)	106(8)	19(9)
LVO detected at PSC (% of AIS)	198(43)	40(69)
LVO detected at CSC (% of AIS)	333(69)	31(67)
Outcomes	**OR (95%CI)**	**OR (95%CI)**
Primary endpoint AIS patients	1.03(0.82-1.29)	1.42(0.72-2.82
Secondary endpoints		
Outcome hemorrhages	0.72(0.44-1.18)	0.94 (0.34-2.63)
Workflow measures	**Minutes (IQR)**	**Minutes (IQR)**
Onset to arrival at PSC (all)	88 (61-145)	82 (65-115)
Onset to arrival at CSC (all)	142(100-231)	116 (95-150)
Time from onset to needle PSC first	120(89-168)	114 (93-157)
Time from onset to needle CSC first	155(120-195)	144 (122-171)
Time from onset to groin PSC first	270(215-347)	222 (196-297)
Time from onset to groin CSC first	214 (172-330)	187 (158-245)
Time from randomization to arrival at PSC	21 (13-32)	28 (21-38)
Time from randomization to arrival at CSC	61 (35-86)	63 (52-71)
Percentage of patients		
Entering within 4 hours at PSC	86,3%	93%
Entering within 4 hours at CSC	76,8%	94%
Wakeup or unknown onset overall	28,6%	1%
Treatment rates		
IVT in PSC-group	60.4%	67%
IVT in CSC-group	47.5%	78%
EVT in PSC-group	39.4%	63%
EVT in CSC-group	48.8%	53%
*Abbreviations: acute ischemic stroke (AIS), large vessel occlusion (LVO), primary stroke center (PSC), comprehensive stroke center (CSC)		
